# Climate, vegetation, people: disentangling the controls of fire at different timescales

**DOI:** 10.1098/rstb.2023.0464

**Published:** 2025-04-17

**Authors:** Sandy P. Harrison, Olivia Haas, Patrick J. Bartlein, Luke Sweeney, Guoxi Zhang

**Affiliations:** ^1^ Leverhulme Centre for Wildfires, Environment and Society, Imperial College London, London SW7 2BW, UK; ^2^ Geography and Environmental Science, Faculty of Science, University of Reading, Reading RG6 6AH, UK; ^3^ Department of Geography, University of Oregon, Eugene, OR 97403, USA

**Keywords:** fire ecology, palaeofires, fire modelling, human influence on fire, fire properties, future fire regimes

## Abstract

Human activities have a major impact on fire regimes. Human activities that cause landscape fragmentation, such as creating roads and other infrastructure or converting areas to agriculture, tend to restrict, rather than promote, fire. The human influence is complex, however, and the impact of fragmentation on the fire regime depends on climate and vegetation conditions. Climate-induced changes in vegetation and fuel loads also affect the natural fire regime in ways independent of human influence. Disentangling the controls of fire regimes is challenging because of the multiple interactions between climate, vegetation, people and fire, and the different timescales over which they operate. We explore these relationships, drawing on statistical and modelling analyses of palaeoenvironmental, historical and recent observations at regional to global scales. We show how these relationships have changed through time and how they vary spatially as a function of environmental and biotic gradients. Specifically, we show that climate and climate-driven changes in vegetation have been the most important drivers of changing fire regimes at least until the Industrial Revolution. Statistical and modelling analyses show no discernible impact of hunter–gatherer communities, and even the time-transgressive introduction of agriculture during the Neolithic had no impact on fire regimes at a regional scale. The post-industrial expansion of agriculture was an important influence on fires, but since the late 19th century, the overwhelming influence of humans has been to reduce fire through progressive landscape fragmentation rather than through influencing ignitions. Model projections suggest that the reduction of fire through fragmentation will be outweighed by climatically driven increases by the end of the 21st century.

This article is part of the theme issue ‘Novel fire regimes under climate changes and human influences: impacts, ecosystem responses and feedbacks’.

## 1. Introduction

Deforestation fires, fires deliberately or accidentally caused by people and so-called megafires receive a considerable amount of media coverage. This, coupled with projections that extreme wildfires will increase by up to 30% by the middle of the century under future climate change [1], has led to a widespread but mistaken belief that wildfires are universally and increasingly directly exacerbated by human activities [2]. Palaeoenvironmental evidence provides information about changing fire regimes in response to the transition from hunter–gatherer communities, through the introduction of agriculture during the Neolithic and subsequent increases in human populations, culminating in post-industrial agricultural expansion. Combining this with direct observations of changing fire regimes during the 20th century provides a more nuanced picture of the influence of people on fire regimes.

Many parts of the world have experienced changes in fire frequency, size and intensity in recent years [3–10]. Model projections suggest that extreme wildfires will increase by up to 30% by the middle of the century [1]. These changes are clearly driven by anthropogenic climate change [11–14]. However, there is considerable concern that other human activities are driving changes in fire regimes. Recent increases in fire, for example, have been attributed to both an increase in human ignitions and to aggressive fire suppression policies [15,16]. This has led to calls for increased intervention and better fire management to mitigate potential deleterious effects on society [17]. However, the recent changes are set against a background of an apparent overall global decline in fire, expressed in recent decades by changes in burnt area largely owing to changes in Africa but with significant declines also in Europe and Asia [18–20]. This decline has been attributed to changes in human land use and the expansion of croplands. Indeed, it is widely recognized that landscape modification by humans is a major influence on fire regimes [21,22].

Part of the confusion about the role of humans arises because fire regimes are influenced by multiple factors, and there are complex interactions between human activities, climate and vegetation [23]. Climate-induced changes in vegetation may affect the natural fire regime in ways independent of human influence, for example through changing fuel loads or fuel wetness, but climate-induced changes in vegetation also influence human behaviour and land use and farming practices [24,25]. Disentangling these different controls is made more challenging because the interactions between climate, vegetation, people and fire operate at different timescales. For example, climate change causes alterations in vegetation productivity on timescales of seasons to years [26,27]; on longer timescales, it influences species distribution through competition [28–30] and can lead to shifts in vegetation type when the climate change is sufficiently large [31,32]—all of which have different impacts on the wildfire regime. The feedback from wildfire to climate also varies with timescale, dominated by changes in greenhouse gas emissions and aerosols or temporary changes in land surface albedo on shorter time scales [33] and by the impact of changes in ecosystem properties on water- and energy-exchanges between the land-surface and the atmosphere in the longer term [23].

A number of strategies have been employed to try and determine the role of people on fire occurrence. There are datasets that provide direct observations of starting time, ignition source and eventual burnt area of individual fires, and these observations are assigned to lightning, human or unknown causes (e.g. [34,35]). However, these direct observations are only available for limited areas and only cover a relatively short period of time. Satellite observations provide information on fire properties globally (e.g. [36,37]), but the registration is weaker at high latitudes. These observations cannot distinguish between human-set and natural fires, although the relative importance of each can be inferred through correlations with human population data or landscape fragmentation indices. However, satellite data only provide a record covering the past few decades. Sedimentary charcoal and chemical records, such as black carbon and ammonium from ice cores, provide records of changes in biomass burning covering many millennia (e.g. [38,39]). Although these records provide no direct evidence for the cause of fires, they can be used to infer the relative importance of climate and human activities by comparing the records with independent data on climate and human population changes. Sedimentary charcoal records indicate a global decline in fire over the whole of the 20th century, which has been attributed to changes in human land use and the expansion of croplands [40], as is the case for the decline in more recent decades. Thus, both palaeo-records and more recent evidence suggest that the most important impact of people has been to reduce fire on a regional to global scale. Modelling also provides a way to evaluate hypotheses about the role of climate and human activities for fire on different timescales.

In this paper, we assess the evidence from all four approaches—direct observations, remote sensing, the palaeo-record and model experiments—for how human activities have influenced fire regimes under modern conditions and in the geologic past before the industrial era. Our goal is to address the following questions. (i) In what way do people influence fire regimes? (ii) How important is human influence on fire regimes compared with other drivers? (iii) How is this influence changing or likely to change in the future? The terminology of wildfire typology is not clear-cut since wildfires could be regarded either as fires in natural vegetation or fires resulting from natural ignition sources. Here, we use the generic term 'fire' unless the fires are clearly set for agricultural purposes or for the purpose of land clearance, and only use the term 'wildfire' for fires that are clearly both naturally ignited and only affect natural vegetation.

## 2. Humans and modern fire regimes

People use fire for specific purposes: for land clearing (e.g. deforestation fires), for pasture improvement, for destruction of pests and weeds in preparing fields prior to cultivation and for burning crop residues [41,42]. Deforestation fires have a significant impact on overall global burnt area and fire-related emissions [43]. Agricultural fires are generally small in extent and have a much smaller impact on burnt area and emissions at a global scale [44]. However, some agricultural fires escape and can generate larger fires in natural vegetation [45]. In addition, humans can cause fires either deliberately through arson or accidentally.

The relative importance of human fire ignitions compared with natural ignitions varies regionally. More than 96% of fires recorded in Europe are ignited by people [46], although most of these are rapidly suppressed. Balch *et al*. [47] indicated that 84% of the total number of fires and 44% of the total burnt area in the conterminous USA between 1992 and 2012 were ignited by people. However, of the fires with known ignition causes in California between 2012 and 2018, 51.7% were caused by lightning and 48.3% by people [48], and lightning-set fires caused a greater proportion of burnt area (56.9% caused by lightning compared with 43.1% caused by lightning). Human ignitions were responsible for 87% of the fires in south-eastern Australia between 1997 and 2009 [49] and a similar breakdown between human (87%) and lightning (13%) was found for the state of Victoria between 2000 and 2019 [50]. However, Janssen *et al*. [51] showed that lightning was the primary cause of fires in extratropical regions and have estimated that 77% of the total burnt area in intact temperate and boreal forests was the result of lightning ignitions.

**Figure 1.**
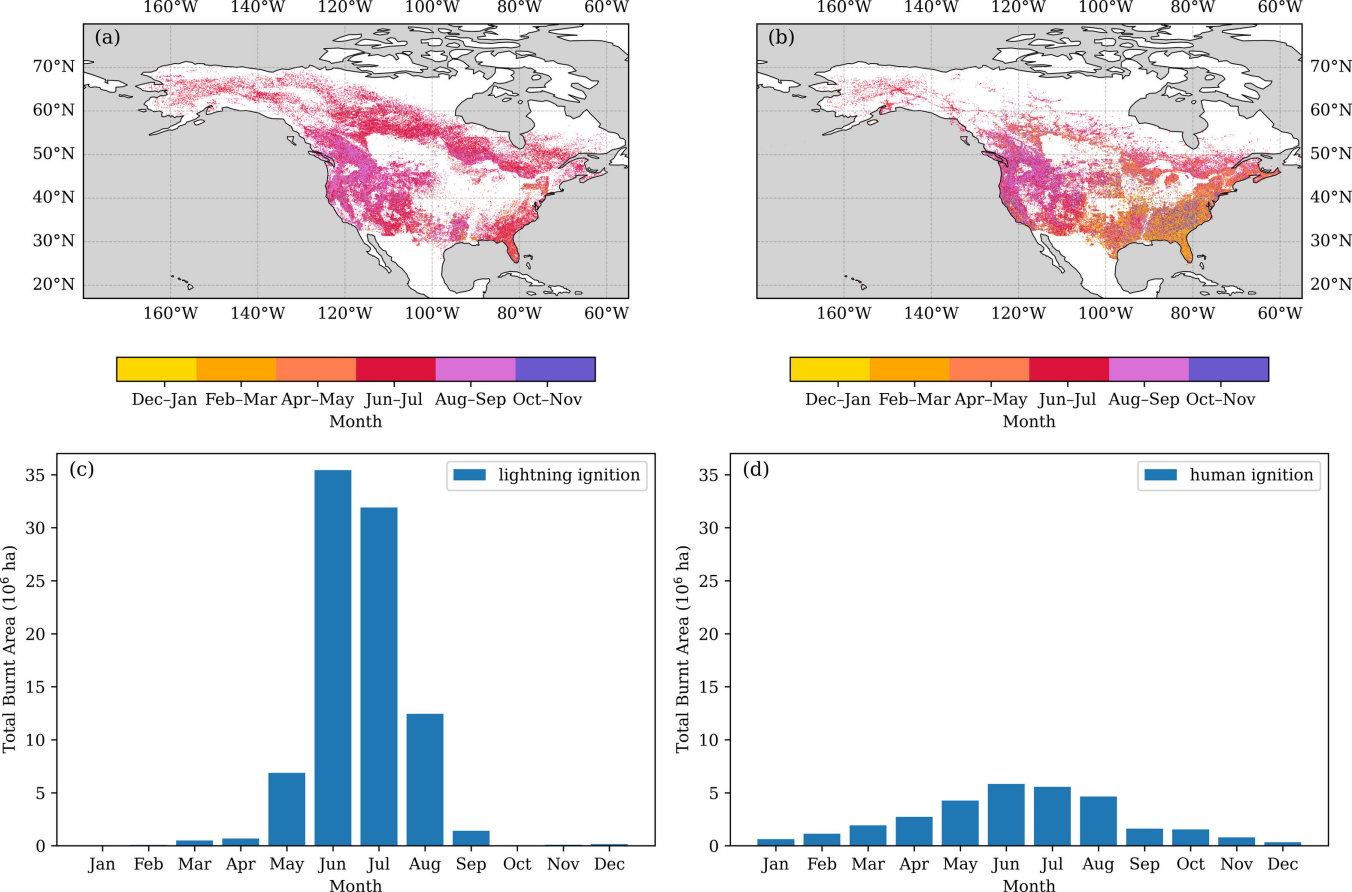
Comparison of seasonal and spatial patterns of fires started by lightning and humans in the USA (https://pages.uoregon.edu/bartlein/FireStarts/index.html; [35]). The figure parts depict spatial patterns of the peak month of fire occurrence of (a) lightning-ignited fires and (b) human-ignited fires, and the seasonality of burnt area of (c) lightning-ignited fires and (d) human-ignited fires.

Fire spread is largely determined by vegetation properties, which determine the availability and continuity of fuel loads, climate factors that influence these vegetation properties, and weather conditions—such as rainfall events and windiness—which affect fuel moisture [52]. Wind strength also has a direct impact on fire spread. These other factors mean that human ignitions only have an impact when the right preconditions exist. The seasonality of lightning-ignited fires across North America ([35]; figure 1a), for example, is broadly mirrored by the seasonality of human-ignited fires (figure 1b). However, the numbers of fires and burnt areas may not provide the best guide to the importance of human ignitions. Although the total burnt area from lightning fires (figure 1c) is much greater (72%) than that from human-set (25%) fires (figure 1d), there is an expansion in the fire season owing to human ignitions, with more fires occurring during the spring (March, April). Balch *et al*. [47] have argued that human-set fires have expanded the fire niche in North America into regions that are wetter and have higher net primary production, which would be consistent with the observed expansion of the fire season (figure 1d) compared with lightning-set fires and with global analyses of the difference in timing between natural and human-ignited fires [53]. The consequences of human-set fires may also be different from those of lightning-set fires. Hantsen and colleagues [48], for example, showed that human-set fires in California spread faster than lightning-ignited fires and produced higher tree mortality and more ecosystem damage—although this might, in part, reflect the fact that these fires generally occurred in areas with lower tree cover and under more extreme fire weather conditions.

Ongoing climate changes are likely to shift the balance between human-set and lightning-ignited fires. The increased severity of fires in recent decades has been attributed to warmer and drier conditions [4]. The impact of warmer and drier conditions on fuel moisture and vegetation flammability has the potential to increase fires in currently fire-free regions (e.g. [54]) and to extend the fire season (e.g. [55]). Furthermore, the amount of lightning is predicted to increase by *ca* 30% per K warming [51,56].

**Figure 2.**
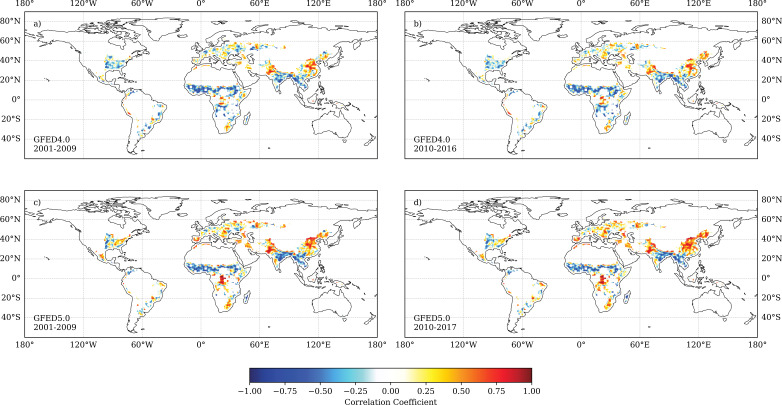
Mapped correlations between burnt area and human population at 0.5° resolution. The population density data are from HYDE 3.2 [60] and have been log-transformed. Grid cells with population values of 0 are masked out. The upper panels show the correlations using burnt area from GFED4.0 [36] during (a) 2001−2009 and (b) 2010−2016. The lower panels show the correlations using burnt area from GFED5.0 [37] during (c) 2001−2009 and (d) 2010−2017. Correlations were calculated using the Spearman rank correlation test.

People also have a role in reducing fires, both through active suppression of existing fires and through landscape management, such as fuel removal or the use of prescribed burning, to reduce the risk or extent of fires [57–59]. However, the biggest cause of human-induced fire reduction is through fragmentation of the landscape by increased appropriation of land for agriculture or infrastructure, which results in increasing fuel discontinuity and thus limits fire spread. Andela *et al*. [18] argued that the observed global decline in burnt area between 1996 and 2015 was driven by socio-economic and land-use changes, primarily in Africa, which increased landscape fragmentation. In general, the amount of landscape fragmentation reflects population density. Figure 2 shows that burnt area is negatively correlated with human population density almost everywhere, a relationship shown in several other studies [61–64]. Exceptions, which show positive correlation between burnt area and population (figure 2), are in densely forested regions, including tropical rain forests in Borneo and south-western Amazonia. There is little difference between the results from the first decade of the 21st century (figure 2a) and the second decade (figure 2b). However, these relationships are shown more strongly in the version 5 of the Global Fire Emissions Data base (GFED5) burnt area dataset (figure 2c,d) than in the GFED4 dataset (figure 2a,b), presumably because GFED5 includes smaller fires that are likely primarily agricultural in origin. Increased landscape fragmentation as a consequence of human population growth has also been proposed as an explanation for the longer-term decline in global fire activity from a peak in the mid-19th century [40,65,66]. The role of landscape fragmentation in suppressing fires is also consistent with the observation that regional decreases in fragmentation, such as that caused by rural abandonment in the Mediterranean, have led to an increase in fires in recent years [67–69].

**Figure 3.**
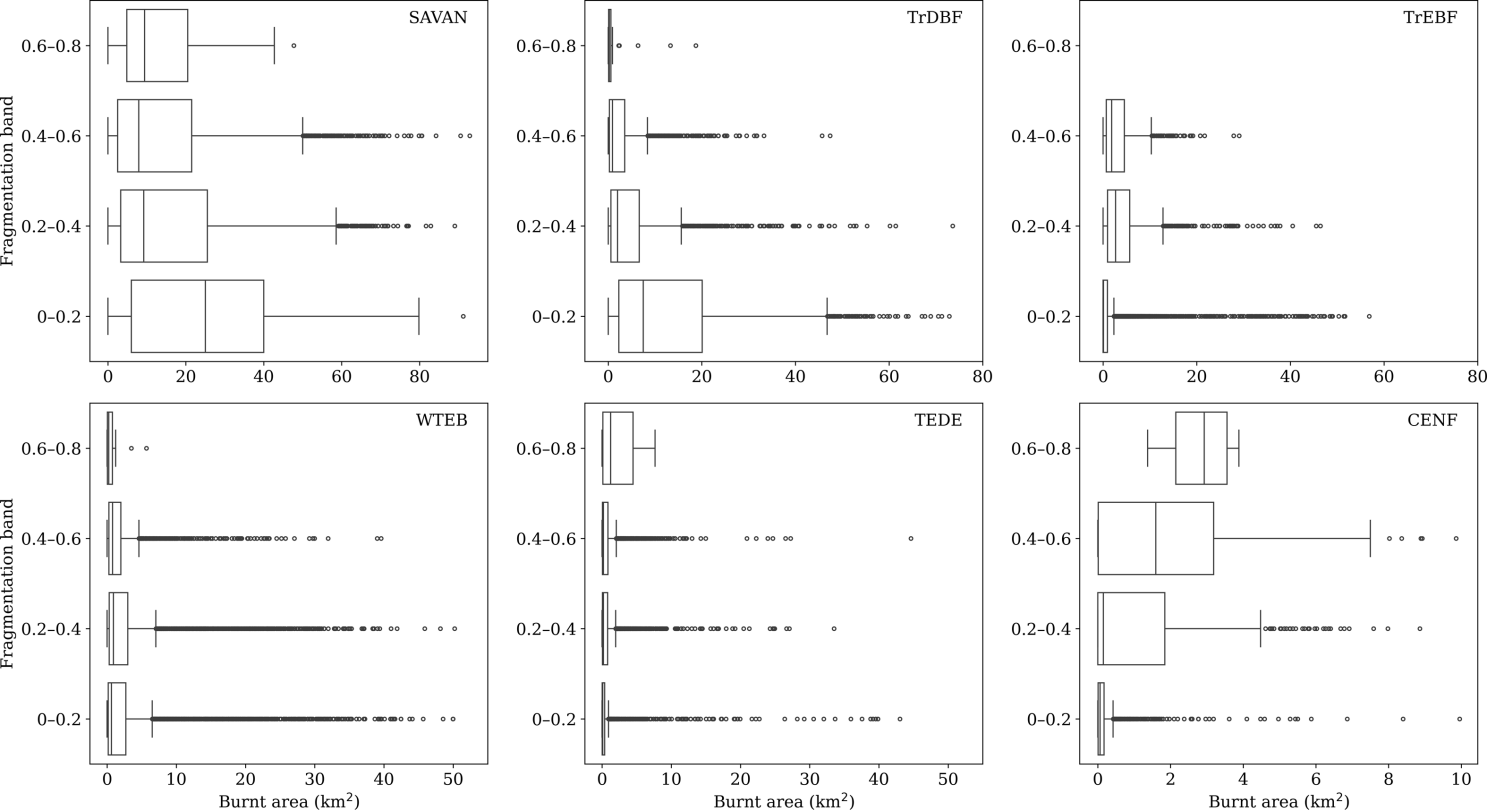
The relationship between fragmentation and burnt area in different forest types. The degree of fragmentation averaged over the period 2001–2017 is taken from the Relative Magnitude of Fragmentation (RMF) dataset (https://portal.geobon.org/ebv-detail?id=4). Bar plots show the degree of fragmentation in different vegetation types against burnt area during the same time interval, taken from GFED5 [37]. The assignment of RMF gridcells to forest types is based on mapped potential natural vegetation [75]. The RMF data are aggregated to 0.25° resolution of the burnt area data. Note that the degree of forest fragmentation never exceeds 0.8 in the RMF dataset. CENF, cool evergreen needleleaf forest; SAVAN, tropical savannah; TEDE, temperate deciduous forest; TrDBF, tropical deciduous broadleaf forest; TrEBF, tropical evergreen broadleaf forest; WTEB, warm-temperate evergreen broadleaf forest.

Nevertheless, the fragmentation of natural ecosystems has different effects depending on whether the vegetation is fire-adapted or not [70]. In ecosystems that are typically relatively fire-free, deforestation, logging and road building create an edge effect that leads to increased wind speeds relative to inside intact forests and also promotes fuel drying, thereby increasing the chance that an ignition will start a fire [71,72]. In contrast, the creation of clearings and roads in typically fire-prone ecosystems interrupts fuel continuity and thus fire spread, leading to a reduction in burnt area [73,74]. This is clearly shown by analysing the relationship between burnt area and the degree of vegetation fragmentation, as given by the Relative Magnitude of Fragmentation (RMF) dataset (https://portal.geobon.org/ebv-detail?id=4), across different biomes. In tropical regions (figure 3, top panels), increasing fragmentation leads to a decrease in burnt area in savannah and tropical deciduous forest ecosystems, which are typically fire-prone; whereas in tropical evergreen forests, which rarely burn under normal conditions, increasing fragmentation is associated with an increase in burnt area. Similarly, in extratropical regions, increasing fragmentation leads to an increase in burnt area in cool evergreen needleleaf forests and temperate deciduous forests, where fires are typically uncommon, but has no impact on overall burnt area in fire-adapted warm-temperate forests, although it causes a decrease in extreme fires.

**Figure 4.**
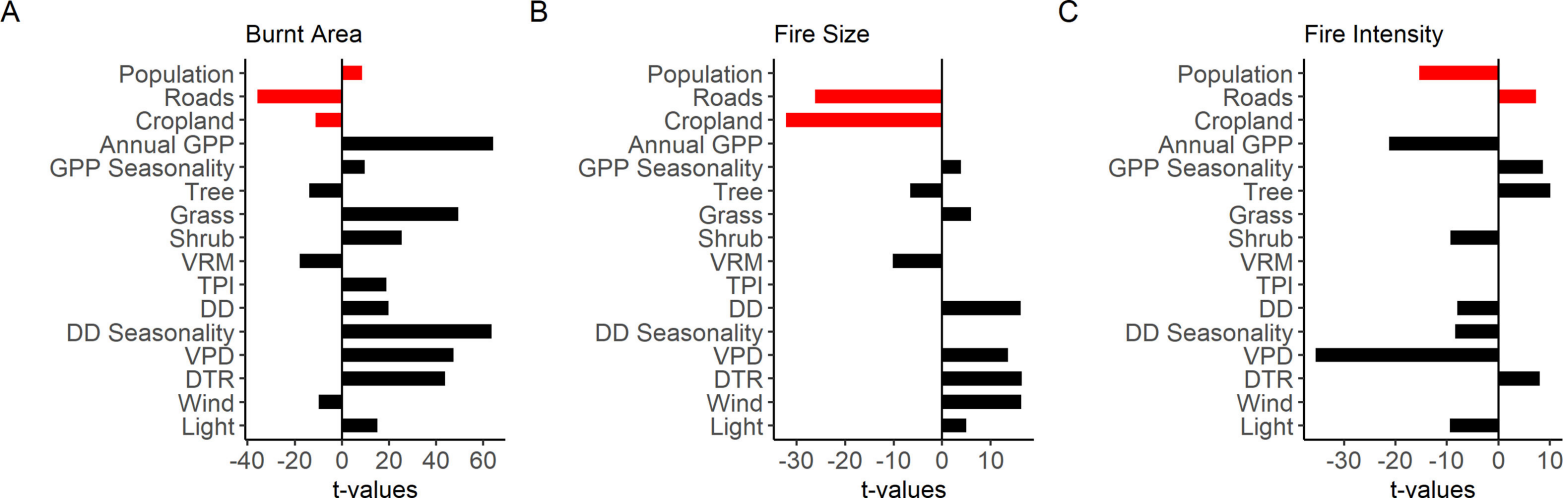
Plots showing the relative importance of factors related to human activities (red bars) compared with other factors (black bars) in explaining (a) burnt area, (b) fire size and (c) fire intensity in three empirical models developed by Haas *et al*. [64]. The human-related explanatory variables are: population density (Population), road density in km^−2^ (Roads) and fractional cropland cover (Cropland); other explanatory factors include annual gross primary production (Annual GPP), the seasonality of GPP (GPP Seasonality), fractional tree cover (Tree), fractional grass cover (Grass), fractional shrub cover (Shrub), two indices of topographic dissection (the vector ruggedness measure (VRM) and the topographic position index (TPI)), maximum number of dry days in a month (DD), seasonality of monthly number of dry days (DD Seasonality), maximum monthly vapour pressure deficit (VPD), maximum mean monthly diurnal temperature range (DTR), mean wind speed of the hottest month in m s^−1^ (Wind), mean monthly lightning ground strikes (Light).

Human activities also have different impacts on different aspects of the fire regime. Empirical modelling [64] has shown that several indicators of human activity, including population density, road density and the area of cropland, are important predictors of global burnt area, fire size and fire intensity alongside factors associated with climate or weather conditions, vegetation properties and landscape topography. However, many of these variables affect burnt area, fire size and fire intensity in different ways (figure 4). Road density, and to a lesser extent crop cover, have a negative impact on burnt area through increasing fragmentation and limiting fire spread. When this effect is accounted for, the residual effect of population density is to increase burnt area. Since road density and crop cover are strongly related to population density, the residual impact of population density is therefore likely to be related to increasing human ignitions with increasing population. This is borne out by the fact that crop cover and road density also both have a negative impact on fire size, but there is no additional effect of population density. Human activities have a very different impact on fire intensity: population density has a negative impact, whereas road density has a positive impact on intensity. The positive impact of road density could plausibly be related to the edge effect in promoting increased wind speeds and drier conditions than in closed forests, and to the correlation between intense deforestation fires and the access roads required in remote forest areas. The negative relationship between fire intensity and population density is likely to reflect the trade-off between the frequency and intensity of fires in regions where fire is used for agricultural purposes, compounded by active fire suppression in more densely populated regions.

## 3. Humans and fire regimes in the past

People have used fire in hunting and as a land management tool for many millennia [76,77], even before the asynchronous shift from hunter–gatherer to agricultural communities during the Neolithic. It has been argued that land-use changes during the Holocene, including changes brought about through the use of fire, had a sufficiently large impact on greenhouse gas emissions to offset the externally forced climate cooling during the late Holocene [78,79]. However, the extent of human-induced changes in land use and fire regimes is highly debated [60,80–82] and the suggested impact of such changes on climate is inconsistent with other lines of evidence about Holocene changes in the carbon balance [83–86]. Furthermore, while using fire for land clearance is harmful to natural ecosystems, traditional pre-agricultural indigenous practices in many regions, including Australia, included burning in the early dry season to minimize the destructiveness of wildfires later in the season [87].

**Figure 5.**
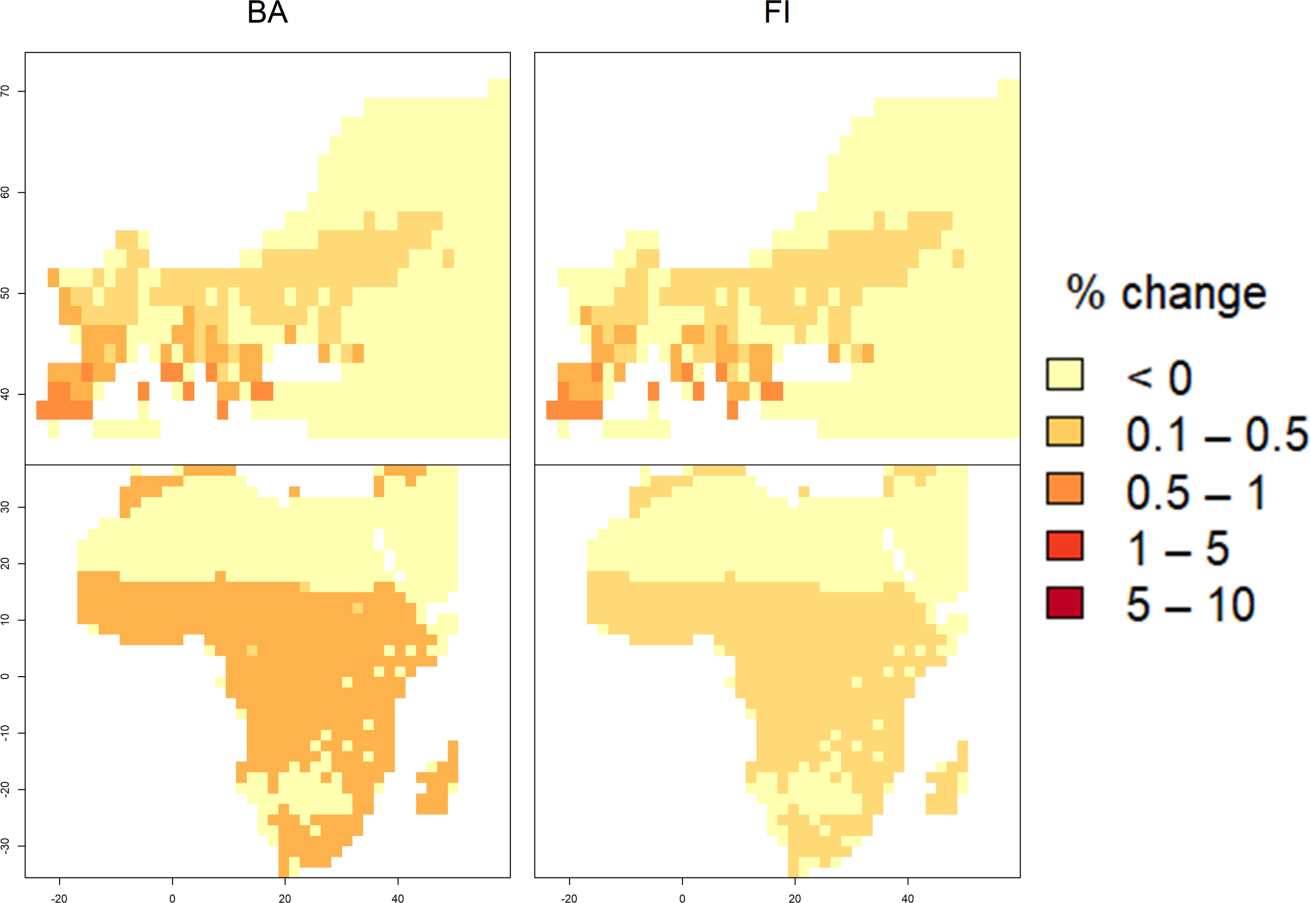
Changes in burnt area (BA) and fire intensity (FI) in Europe and Africa as a result of including human activities in simulations of fire at the Last Glacial Maximum (*ca* 21 000 years ago). The simulations were made with empirical models of BA and FI driven by climate, vegetation properties, topographic factors and indices of human activities, including population density and road density [64]. The LGM climate was obtained as an ensemble average of three earth system models; vegetation properties were then simulated using a light-use efficiency model for GPP and a couple of biogeography–biochemistry models, and atmospheric CO_2_ was obtained from ice-core records. The empirical models were first run after setting human factors to zero and then re-run using estimates of population density based on archaeological information. Full details on the results of these experiments are given in Haas *et al*. [98]. The plots here show the percentage increase in BA and FI as a result of including estimates of human population for these two regions taken from Tallivaara *et al*. [97] and Gautney & Holliday [96], respectively, compared with a simulation in which the influences of human activities on fire regime were not included.

Palaeoenvironmental evidence can be brought to bear to address past changes in fire regimes at regional or global scales. Burning vegetation produces charcoal, black carbon and carbon spherules that accumulate naturally in lake sediments, peat, soils and marine sediments [88]. Sedimentary records can therefore be exploited to provide a record of changing fire activity at individual locations at a temporal resolution ranging from annual—in laminated lake sediment—to centennial in sites where sediment accumulates more slowly. Although charcoal records do not provide quantitative estimates of biomass burnt or burnt area, they can be interpreted in terms of relative changes in biomass burning at regional to global scales (e.g. [66,89–93]). The Reading Palaeofire database [39] currently provides 1676 individual records from 1480 sites worldwide, and covering all continents except Antarctica. Although the temporal coverage is best for the past few thousand years, there are records covering the last glacial cycle, including intervals of rapid climate change typified by the Dansgaard–Oeschger (D–O) warming events in Greenland [94]. Analyses of these records have shown that fire has tracked millennial-scale climate variability during the last ice age [89–91], with global increases in fire during the D–O warming events in Greenland and reductions in fire during the cold periods characterized by Heinrich stadials in the North Atlantic. The Last Glacial Maximum (LGM, *ca* 21 000 years before present) was characterized by low biomass burning over most of the world [92], consistent with the colder and drier conditions experienced at this time. The area that could support people was reduced, and population densities even in these regions were low [95–97]. Simulations with empirical models of burnt area and fire intensity [64] driven by changes in climate, vegetation properties and atmospheric CO_2_ concentration at the LGM show that the inclusion of estimates of hunter–gatherer populations was insufficient to have any noticeable impact on fire regimes [98]. Even in Europe and Africa (figure 5), which archaeological records indicate had the highest population densities at the LGM [96,97], the increase in burnt area and fire intensity as a result of human activities is always well below 5%. Charcoal records show a gradual global increase in fire during the last deglaciation, but the changes were asynchronous between the northern and southern hemispheres although consistent with the long-term temperature trends shown by ice-core records in Greenland and Antarctica, respectively [92].

**Figure 6.**
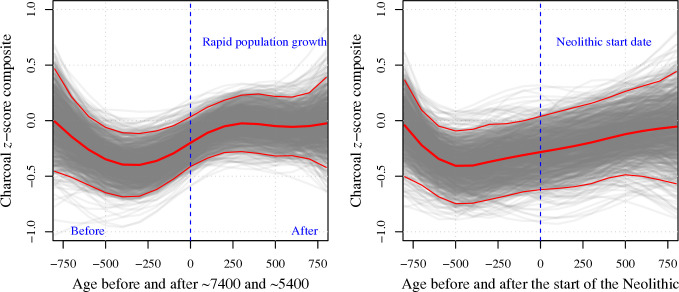
Comparison of fire history and human activities in Iberia during the Holocene, redrawn from Sweeney *et al*. [93]. The left plot (a) shows a superposed epoch analysis of the composite charcoal influx across sites in Iberia during two intervals of rapid population growth, ca 7400 and 5370 years before present. The right plot (b) shows the composite charcoal influx before and after the start of agriculture at each site. The solid red line in each plot shows the median *z*-score value of the charcoal and the fine red lines show the 95% confidence intervals; the grey lines represent 1000 bootstrap resamples of the site data.

Human impacts on fire regimes are expected to become more noticeable during the Holocene, with the spread of agriculture. Records from individual sites often show increased charcoal accumulation at times when other indicators of human modification of the landscape also increase (e.g. [99]). However, these correlations are not necessarily causal since human use of the landscape is also conditioned by climate. Regional comparisons often fail to show strong correlations between changes in population or human activities and fire regimes. Haberle & Ledru [100], for example, compared charcoal records from Central and South America and Indonesia and Papua New Guinea with climate data and information about human occupation; they found limited evidence of synchrony between changes in fire regimes and human activities but a much stronger synchrony between the charcoal records and climate changes. Marlon *et al*. [66] examined the relationship between changes in charcoal and reconstructions of human population density for multiple regions across the world and again found little evidence of synchrony. A more detailed analysis of Holocene charcoal and archaeological records from the Iberian peninsula [93] showed no correlation between regional changes in charcoal abundance with either intervals of rapid population growth (figure 6a) or archaeological evidence for the beginning of Neolithic agriculture at individual sites (figure 6b).

Nevertheless, the most recent part of the charcoal record documents human impacts, specifically a global increase in fire after ca 1750 CE, coincident with population expansion during the warming after the Little Ice Age and reaching a maximum around 1850 CE and a subsequent decrease in fire towards the present day [40]. Since the decrease in fire pre-dates the adoption of active fire fighting in most regions of the world, Marlon *et al*. [40] argued that this provides evidence for the importance of landscape fragmentation in suppressing fire.

## 4. Humans and future fire regimes

**Figure 7.**
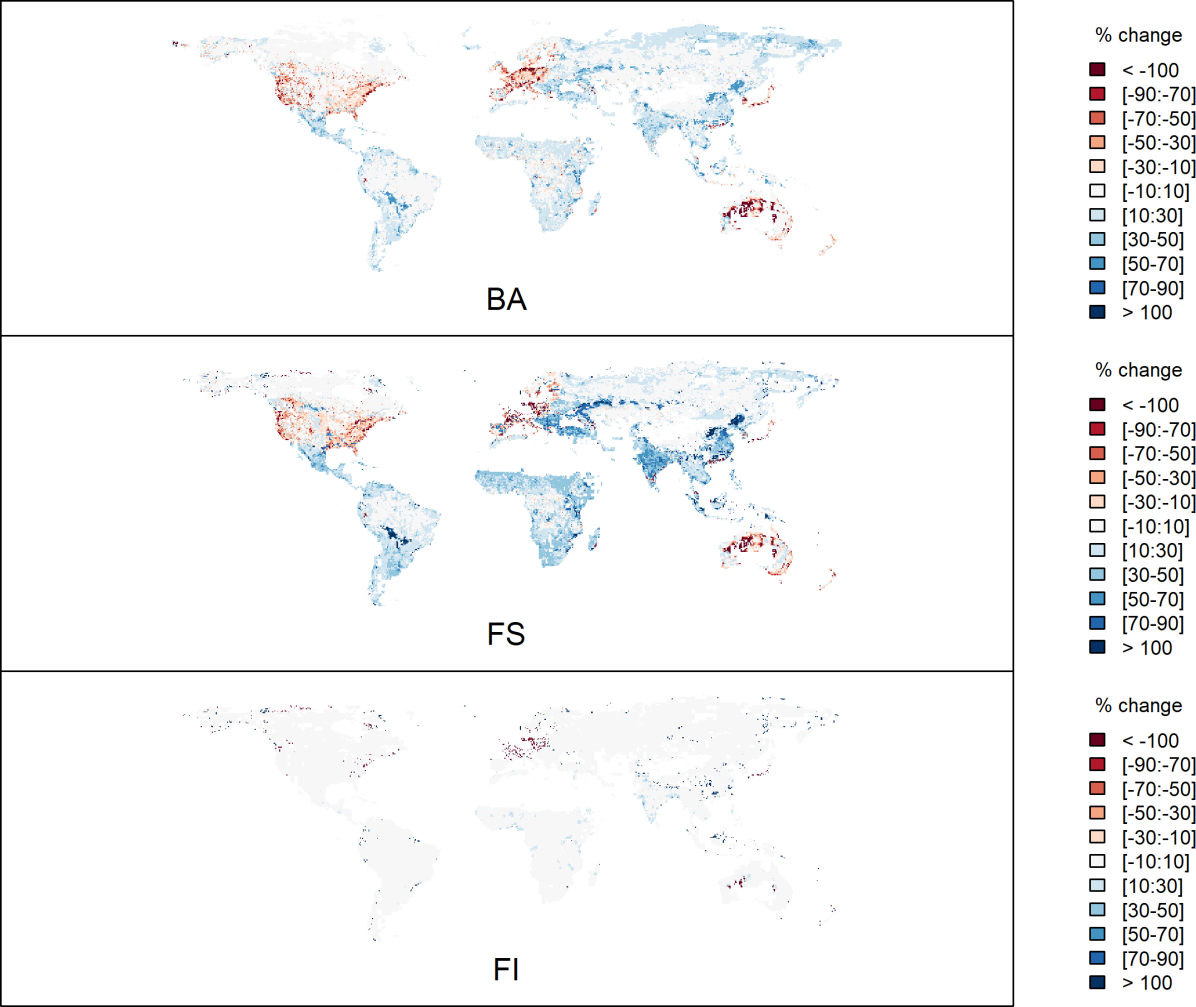
Simulated differences in burnt area (BA), fire size (FS) and fire intensity (FI) by 2100 CE under a low-mitigation climate scenario (RCP 6.0) using two different socio-economic scenarios—the SSP1 sustainable development scenario and the SSP3 regional inequality scenario—to illustrate differences owing to human activities.

**Figure 8.**
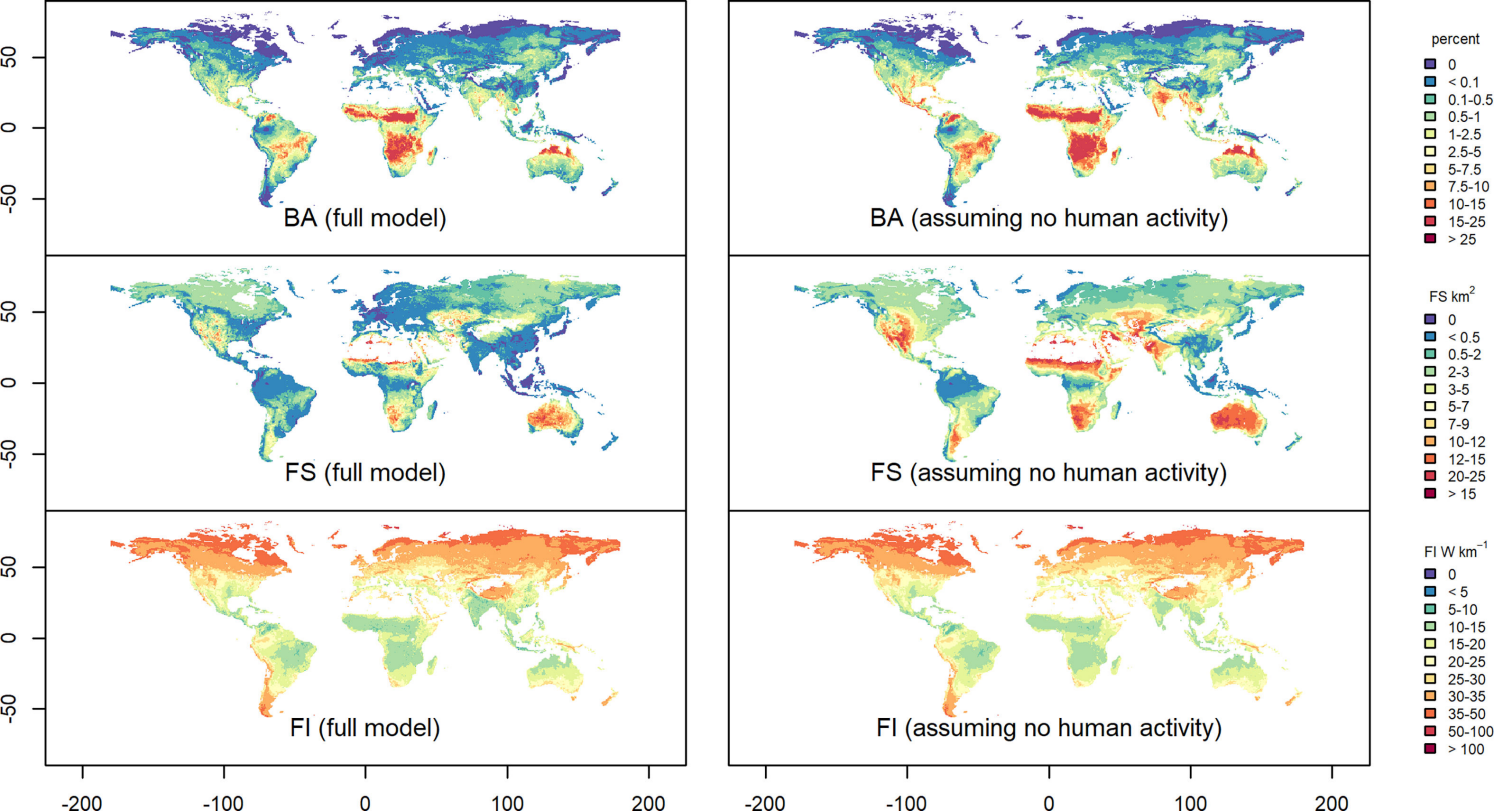
Impact of human activity on fire regimes under modern conditions shown by the difference in burnt area (BA), fire size (FS) and fire intensity (FI) between a baseline simulation that includes human drivers (full model) and a counterfactual experiment when these drivers are set to zero (assuming no human activity).

Climate change and changes in human activities are expected to have a major impact on future fires. Weather conditions promoting increased fire risk consistently increase in future climate projections [14]. Simulations with fire-enabled dynamic vegetation models suggest that extreme fires will increase by up to 30% by the middle of the current century [1]. Empirical modelling shows that the impact of climate change on fire regimes, though strongly dependent on the climate scenario used to drive the simulations, differs substantially between tropical and extratropical regions [101]. Under the low-mitigation scenario, climate and vegetation changes lead to a 56% increase in burning, with larger and more intense fires, in the extratropics over the 21st century. Fires also expand into regions that rarely experience fire today. In tropical regions, burnt area is reduced by 19% in high-mitigation climate scenarios through human activities, but in low-mitigation scenarios, the climate changes overwhelm this signal and fire increases substantially (27%). However, different scenarios of human activity could impact these patterns. Comparison of simulated changes under Representative Concentration Pathway (RCP .5) but using two different socio-economic scenarios—the Shared Socioeconomic Pathway (SSP) sustainable development scenario and the SSP3 regional inequality scenario—shows substantial differences in fire regimes by the end of the 21st century with an amplification of the divergent trends in the tropics and extratropics (figure 7). In western Europe, for example, lower population densities under SSP3 compared with SSP1 cause an increase in burnt area and fire size. In North America, the larger reduction in the area of cropland under SSP3 compared with SSP1 leads to an increase in burnt area and fire size (figure 7). However, major increases in population, cropland area and road density in other regions under the SSP3 scenario lead to a decrease in burnt area, fire size and fire intensity. The impact of different future scenarios of human activity is congruent with counterfactual model simulations of the present day showing that removing the influence of human activities leads to increased fire size and burnt area but has little discernible effect on fire intensity (figure 8).

## 5. Discussion and conclusions

There is a disconnect between public perception of the role of humans in exacerbating wildfire regimes and the observational evidence showing that human activities tend to reduce the occurrence and severity of wildfires. There is little concrete evidence (table 1), for example, that changes in human activities associated with the time-transgressive introduction of agriculture during the Neolithic led to regional increases in biomass burning during the Holocene. However, post-industrial agricultural expansion in the 19th century appears to have caused an increase in biomass burning globally, with the biggest impacts registered unsurprisingly in North America [40]. Both palaeo-records, historic records and more recent satellite-based evidence suggest that the most important impact of people has been to reduce fire on a regional to global scale, although it has been suggested that uncertainties in the observations may have caused the scale of this impact to be exaggerated [20]. Nevertheless, regional declines in burnt area over the past four decades are statistically significant in northern and southern Africa, central Asia and Europe, while there are no statistically significant increases in burnt area in other regions [20]. These recent declines in burnt area are also associated with declines in the number of fires and fire size [18] and are associated with a decline in fire-related pollutants (PM2.5), at least at a regional scale [102]. The importance of landscape fragmentation in reducing fire occurrence is borne out by empirical analyses, which show that increasing population density only has a positive impact on burnt area when other measures of landscape fragmentation are included in the model [64]. However, model projections of future fire regimes suggest that the influence of landscape fragmentation may be overwhelmed by the impact of climate changes during the 21st century [101].

**Table 1.** Summary of impact and relative importance of climate (and climate-induced vegetation changes) compared with human impacts on fire regimes during different time periods at a global scale, based on observations and model analyses. The human impacts are considered in terms of the impact of population changes on ignitions, the impact of landscape modification and the indirect impact of landscape fragmentation. The time periods are the rapid warming events during the past ice age (Dansgaard–Oeschger, D–O, warming), the colder and drier climate of the Last Glacial Maximum (LGM), warmer summers in the northern hemisphere during the mid-Holocene (an interval broadly coincident with the introduction of agriculture during the Neolithic), the northern hemisphere summer cooling during the last Holocene, the post-industrial expansion of agriculture (1750−1850 CE), the 20th century and the satellite-observation period of the past four decades. Impacts leading to an increase in fire are shown with a + and a decrease with a −, where the number of symbols gives an indication of the relative magnitude of the impact and (+) means the effect is present but very small. There is no evidence for some impacts at certain times, indicated by *ne*.

	climate	human impact		
		ignitions	landscape modification	fragmentation
D–O warming	+++	*ne*	*ne*	*ne*
LGM	—	(+)	*ne*	*ne*
mid-Holocene	+++	*ne*	*ne*	*ne*
late Holocene	*—*	*ne*	+	*ne*
1750−1850 CE	+	++	++	*ne*
20th century	*ne*	*ne*	—	—
past four decades	+	+	+	—

Given that both wildfires and agricultural fires have negative impacts on atmospheric pollution (with feedback to climate [33,103]), human health and wellbeing [104–106], human infrastructure and economy [107–109] and ecosystem services [110–113], active wildfire management may become increasingly necessary [114–116]. However, the adoption of integrated fire management (IFM; [117,118]) solutions needs to be based on balancing the risks and benefits of wildfires, particularly given the fact that many ecosystems are adapted to or maintained by wildfires. Model experiments in which wildfire is completely suppressed show large increases in tree cover causing transitions from savannahs and tropical dry woodlands to forest [70,119,120], a result consistent with impacts of fire exclusion experiments at a local scale [121]. The success of IFM will also be dependent on a more realistic understanding of the relationship between people and fire.

## Data Availability

The data used are all available from public repositories and we have provided the details to access these data in the text.
